# Advancing dementia care in Pakistan: challenges and the way forward

**DOI:** 10.3389/frdem.2023.1241927

**Published:** 2023-09-14

**Authors:** Soha Ali, Maha Zehra, Tehreem Fatima, Abdullah Nadeem

**Affiliations:** Department of Medicine, Dow University of Health Sciences, Karachi, Pakistan

**Keywords:** dementia, dementia care, Pakistan, degenerative brain disease, Alzheimer's disease

## Abstract

Dementia encompasses a wide range of cognitive and psychological impairments that hinder individuals' ability to carry out daily tasks effectively. In the context of Pakistan, the prevalence of dementia patients currently stands at ~150,000–200,000, reflecting the impact of the country's significant population size. This increase in numbers poses a substantial socioeconomic challenge, emphasizing the need to prioritize dementia within Pakistan's healthcare system. However, the allocation of resources and attention to dementia remains relatively low, leading to considerable difficulties in both diagnosing and treating affected individuals. The provision of comprehensive dementia care faces numerous obstacles, including limited public awareness, insufficient research initiatives, inadequate infrastructure, and a lack of specialized training programs. To address these challenges, the Pakistani government must acknowledge and address the stringent regulations governing the neuroscience industry, with a specific emphasis on catering to the unique needs of dementia patients. By doing so, they can ensure the delivery of high-quality care, essential support, and necessary resources for individuals living with dementia in the country.

## Introduction

Dementia is not a specific disease but rather a condition that encompasses a group of symptoms, including but not limited to a reduction in memory capacity, alterations in cognitive abilities, impaired judgment and reasoning, diminished concentration and attention, modifications in language usage, and changes in behavioral patterns. It arises from the degeneration or impairment of neuronal cells and their synapses within the brain. The manifestation of dementia and its associated symptoms can vary depending on the specific region of the brain that has been affected (Mayo Clinic, 2023). The WHO estimates that there are over 55 million individuals worldwide affected by dementia, with over 60% of them living in low- and middle-income nations. There are roughly 10 million novel occurrences per year, with Alzheimer's disease type (DAT) responsible for 60–70% of total cases (Dementia, 2023). In DAT, there is a progressive deterioration in psychological and cognitive functions. It is becoming a major concern in the medical, social, and economic domains since the incidence and prevalence are rising with the increasing population of older people. The occurrence of DAT has been subject to numerous investigations worldwide, revealing a notable trend of increasing frequency. The estimated prevalence of all-cause dementia among individuals aged 50 and over in the community was 697 per 10,000 persons, based on the meta-analysis of forty-seven studies conducted between January 1985 and August 2019 (Cao et al., 2020). Furthermore, between 1990 and 2019, there was a significant rise in the incidence and prevalence of Alzheimer's disease and other dementias, with the incidence increasing by ~147.95% and the prevalence by around 160.84% (Li et al., 2022). This substantial increase highlights the growing impact of these cognitive disorders on global health during that period.

**Figure 1:**
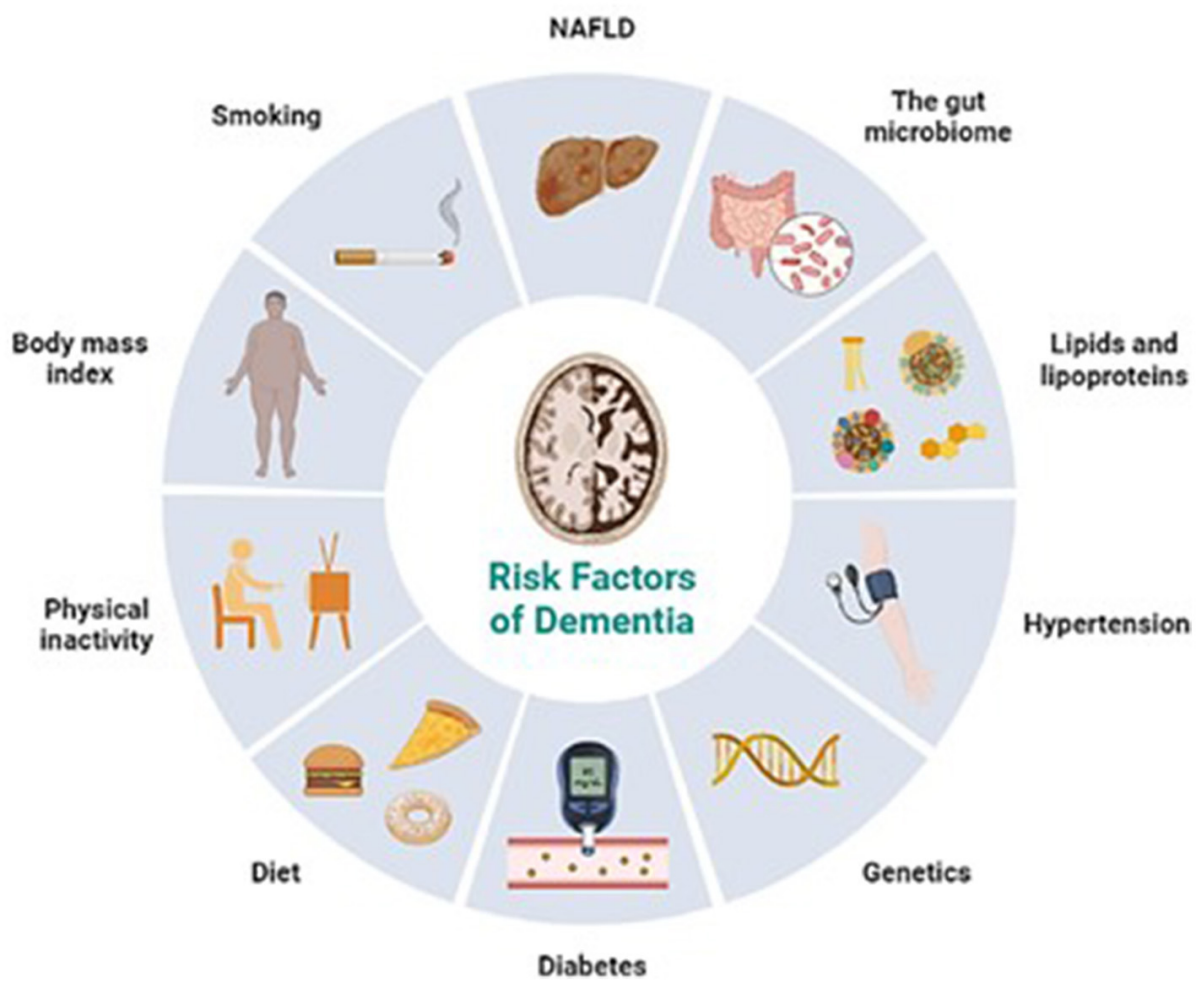
Risk factors of dementia (Retrieved from https://app.biorender.com/biorender-templates).

The amyloid-beta peptide (Aβ) accumulation in brain tissues is associated with developing neuritic plaques and neurofibrillary tangles, a pathological characteristic of Alzheimer's disease. These structures primarily affect the medial temporal lobe and associative neocortical structures (De-Paula et al., 2012). As mentioned earlier, it is hypothesized that these aggregates have damaging effects on the survival of normal neurons and the axonal projections that link them. The risk factors of dementia are shown in Figure 1. While a cure for dementia remains obscure, various interventions can be employed to support individuals living with the condition, who will need additional assistance and care as the disease worsens to effectively control their symptoms. Ongoing research is continuously revealing new insights into the complex and multifaceted pathology behind Alzheimer's disease, as well as other types of dementia. As scientific understanding advances, it becomes increasingly evident that dementia's origins lie in intricate biological processes that demand further investigation and exploration.

Pakistan is the sixth most populated in the world, with over 150,000 people reported to have dementia. According to projections, the population of individuals aged 65 and above is anticipated to rise from 8 million to 27 million by the year 2050, thereby augmenting the prevalence of dementia (Ahmad et al., 2013).

## Challenges of dementia care in Pakistan

Pakistan, being a third-world country, is up against many obstacles when providing dementia care. Dementia is unfortunately given low priority in the healthcare system of Pakistan. Studies have shown the public has an unsatisfactory understanding of dementia. It is frequently perceived as a typical outcome of aging by many individuals. Early diagnosis is further hindered by a general lack of knowledge about accessible public healthcare options available to them (Balouch et al., 2021). Moreover, it is culturally stigmatized for families to use residential care, as the care of old people is culturally and religiously viewed as the complete responsibility of their families.

The current state of infrastructure in Pakistan poses a challenge for clinicians and researchers working in the field of dementia. There simply is not enough research and published literature on dementia. Presently, 1,086 clinical trials on Alzheimer's disease are in the works worldwide, yet none of these are being conducted in Pakistan (Khan, 2014).

As previously stated, there is currently no treatment for dementia, although established support services can assist persons with the condition (Somme, 2010) and their caregivers in the family to live better lives (Downs et al., 2008; Farina et al., 2017). It is therefore imperative that patients get an early diagnosis and have access to services that could mitigate the situation. Unfortunately, good public health centers, long-term care facilities, and specialized residential care in Pakistan are scarce, or in most cases, almost non-existent (Zaidi et al., 2018) in most individuals being cared for by their families in a home setting (Khan, 2014). These are not enough to serve the entire nation. Additionally, it is unknown if the few general care facilities in Pakistan are aware of the actual percentage of their residents who may have dementia (Leroi et al., 2019). This can naturally lead to increased chances of mismanagement and harm.

There is barely any formal support available for caregivers. At the primary care level, social and economic background further comes into play: well-off families may have access to information and necessary resources such as nurses, while middle or low-income households must rely entirely on familial and other informal care resources, for example, domestic helpers (Zaidi et al., 2018). This places undue pressure on the familial caregivers, not to mention the risk of endangering patients as they are being looked after by untrained home-based care.

Even in neurology training, dementia is not given the proper priority, despite being a serious health concern (Khatri and Wasay, 2013). There is a lack of dementia training programs available for neurology residents and an absence of neuroscience training programs at both the undergraduate and graduate levels. Similarly, there are no postgraduate training courses in geriatric medicine. In addition to neurologists, psychiatrists, too, often face a similar challenge, as there is a notable shortage of dementia training in their field, leaving many healthcare professionals ill-equipped to manage and address the specific needs of individuals with dementia. The curriculum of the few training programs that are being taught in Pakistan remains insufficient (Leroi et al., 2019). Relevant and crucial information on prevention, diagnosis, and management is practically absent.

Treatment provision and diagnosis have sadly not been able to keep up with the incidence of dementia. According to a survey that was published, there are only 35 neurosurgical clinics and 1 neurosurgeon for every 1.37 million people in the nation (Khan et al., 2014). Dementia care, due to geriatric medicine not being a separate distinct subspeciality, falls under the umbrella of neurology and psychiatry. Taking that into consideration, alarming statistics have been published by Khan et al. (2014). There is just one dementia registry, one research center, one trained specialist, one day-care center, and two academic research clinics in the entire country (Khan, 2014). This is disproportionate to the requirements of the population.

For evaluating dementia and associated risk factors, such as neuroimaging of brain damage and aging, state-of-the-art technology is significantly insufficient, as are resources including specialty-trained doctors, research and educational initiatives, and public awareness campaigns. The fact that Pakistan rates highly for risk factors that might result in brain injuries, such as a lack of road regulations, domestic and sexual violence, and terrorism, makes this situation worse (Adamson et al., 2020). Fortunately, acute care services are being provided, for example readily available ambulance networks (Chotani, 2002; Razzak et al., 2015). There is no proper system established to carry out the necessary follow-ups and rehabilitation, for which there is a need for a detailed patient registry.

Together with Shifa International Hospital in Islamabad, Alzheimer's Disease International is developing a dementia registry and national dementia guidelines (Adamson et al., 2020). However, there aren't any biobanks, database protocols, or any focus on community outreach. At present, Pakistan lacks a governmental policy offering specialized healthcare services to individuals who have dementia. Unfortunately, both resources and funding are severely limited. There is a need for improved infrastructure to enable effective work in this area and to extend essential support to patients (Khan, 2014).

## Existing efforts and initiatives

The country has an incredibly poor record of addressing social and economic issues pertaining to older people. The bulk of the nation's health science programs does not even concentrate on aging-related brain health problems. The Sussex Institute of Neuropsychiatry in Islamabad, however, is one new organization that is developing courses to tackle this issue (Leroi et al., 2019).

Currently there is no government policies set in place, and there is little to no evidence that any such future initiative is in the works (Zaidi et al., 2018). In 2019, “Roadmap for Dementia Research in Pakistan” was developed by international expert stakeholders as a model for all LMICs. The goal was to set principles and priorities for developing research, thereby leading to national policies and systems. These focus areas were derived from the WHO's “Global Action against Dementia,” which arose from the 2013 (Shah et al., 2016; G8 Dementia Summit Declaration, 2023). The initiative has triggered significant advances in research, but the situation in LMICs continues to be the same.

Relevant research, evidence-based policymaking and resources needed to deal with this rising epidemic are limited (Qadir et al., 2013; Khan, 2014). Pakistan receives a shockingly low ranking in the Global Age Watch Index (Zaidi et al., 2019), especially when it comes to the wellbeing of senior citizens. Their comparatively short lifespans and even shorter healthy lifespans shed insight into the condition of the aforementioned people. An in-depth study on dementia has been conducted in LMICs by the renowned 10/66 Dementia Research Group. Unfortunately, Pakistan was not one of its research locations (Prince, 2010).

There is a deficiency of health professionals who are willing to get involved in research related to non-communicable diseases (NCDs) such as dementia and mental health (Thornicroft et al., 2012; Huffman et al., 2015). No documented longitudinal cohort research or randomized controlled trial (RCT) for dementias like DAT has been conducted in Pakistan (Leroi et al., 2019). The Higher Education Commission (HEC), a national organization that regulates all facets of higher education and research in Pakistan, has examined the problem and recommended expanding international cooperation and updating the existing funding systems for supporting research (Nadeem et al., 2023).

As long as the policies put in place are sympathetic to cultural differences, the knowledge transfer from research done elsewhere in the world can help the nation overcome its lack of literature (Zaidi et al., 2018). A study has argued that South Asians experience similar challenges in accessing mental health services in different countries, thereby making the literature from one country applicable to another (Giebel et al., 2015). According to the study by Zaidi et al. (2019) at-home solutions need to be developed since home-based interventions are preferred country-wide. Hence, assistive technology is being created and applied to overcome the barrier to service allocation, for example, the Assisted Brotherhood Community (ABC) application which showed promising results (Asghar et al., 2020).

A daycare center has been established in Lahore, Pakistan, through the partnership of Alzheimer's Pakistan, a private organization, and Alzheimer's Australia. It allows people with dementia to stay during the daytime only (Balouch et al., 2021), there is just one local hospital in Karachi that offers various support services to aid in the management of the condition. Support groups and the necessary instructional workshops for families and unofficial caregivers of dementia patients are beginning to appear, but not on a nationwide scale. A properly structured training program for caregivers has recently been introduced by Alzheimer's Pakistan in collaboration with Alzheimer's disease international. Even though the impact is positive, it is still far from what is required.

## Proposed strategies for the way forward

It is necessary to remove barriers to the way dementia patients receive healthcare to enhance care pathways and raise public awareness. Starting public awareness initiatives through TV, radio, and social media emphasizing early symptom recognition, prevalent myths, appropriate care, affordable healthcare facilities, and avoidance strategies is necessary. In addition, seminars should be regularly scheduled to strengthen healthcare providers' capacity in treating dementia. Postgraduate programs for training in behavioral neurology and geriatric psychiatry would be beneficial. National dementia screening schemes need to be established. Healthcare centers should have designated inpatient wards with sufficient amenities and supplies to serve dementia patients (Gale et al., 2018). As promising treatments like lecanemab and donanemab demonstrate potential therapeutic breakthroughs in dementia management, it becomes imperative to anticipate and plan for the potential future need for increased facilities and healthcare settings that can effectively administer and provide these innovative treatments to a broader population of individuals with dementia.

Hiring housekeepers specifically trained to care for individuals with dementia, in addition to or instead of family members or health professionals, can significantly enhance patient outcomes and enable them to continue living at home. These formally educated housekeepers possess specialized knowledge and skills to effectively support and attend to the unique needs of dementia patients. With their expertise, they can provide personalized care, manage challenging behaviors, and maintain a safe and comfortable environment for the individual. The initiative can be set up using educational courses for in-home providers. An example of such an initiative would be changing dementia through research and education by the US Alzheimer's association. In the cultural context of the nation, these resources can be translated into regional languages. Seminars for training can be facilitated by nurses with geriatrics experience. Housekeepers who currently or want to provide care for the elderly would be the population of interest (Thaver and Ahmad, 2018). This approach not only improves the quality of life for the individual but also ensures that they receive tailored and continuous care, maximizing their ability to remain in a familiar setting where they feel most comfortable.

Many countries have recognized the need for a comprehensive and coordinated approach to dementia care, which involves various stakeholders and goes beyond the medical model. Some examples of national strategies from different countries that focus on multidisciplinary collaboration and addressing stigma are listed below:

1 United kingdom - 10-year plan
In 2019, the UK government published the “Dementia 2020 Challenge,” a 10-year plan aimed at improving dementia care and support. The plan emphasizes five key priorities: awareness and societal understanding, improving dementia diagnosis rates, providing better support for those living with dementia, supporting the dementia workforce, and promoting research to improve care and find a cure. It highlights the need for a person-centered approach involving health and social care professionals, along with family and community support. The plan also aims to reduce the stigma around dementia through public awareness campaigns and community engagement (Health Secretary Announces 10-year Plan for Dementia, 2023).
2 United states - national plan 2022 update
The United States' “National Plan to Address Alzheimer's Disease” was first launched in 2012 and has been regularly updated. The 2022 update emphasizes the importance of early detection and diagnosis, supporting caregivers, and advancing research for effective treatments and prevention. The plan encourages a collaborative effort between the public and private sectors, healthcare providers, and community organizations to address the challenges posed by Alzheimer's disease and related dementias. It also prioritizes improving the quality of care provided to individuals with dementia and enhancing the overall dementia-friendly environment (National Plan to Address Alzheimer's Disease, 2022).
3 Australia - national dementia plan
Australia's “National Dementia Strategy” outlines a comprehensive approach to dementia care and support. It focuses on five strategic priorities: dementia-friendly communities, accessible information and services, quality care and support, research and innovation, and a well-supported and skilled workforce. The strategy aims to improve the quality of life for people with dementia, reduce stigma, discrimination, and increase awareness and understanding of dementia in the community. It emphasizes partnerships and collaboration between various sectors, including health, aged care, and community services (Australian Institute of Health and Welfare, 2023).
4 The Netherlands - national dementia program
The Netherlands has a national dementia program known as “*Deltaplan Dementie”*. The program focuses on three pillars: research, care, and social participation. It aims to improve dementia care and support through early diagnosis, specialized care facilities, and initiatives to promote social engagement and inclusion for people with dementia. The program encourages collaboration between healthcare providers, social workers, municipalities, and research institutions. It also addresses stigma and raises public awareness through educational campaigns (Netherlands Alzheimer Europe, 2023).
5 Sweden - national dementia strategy
Sweden's national strategy for dementia centers on three main goals: improving the quality of life for people with dementia, supporting their family members and caregivers, and promoting research and knowledge dissemination. The strategy emphasizes a person-centered and rights-based approach to dementia care. It focuses on providing specialized training for healthcare professionals, enhancing community support services, and implementing dementia-friendly practices in various settings. The strategy also addresses stigma through awareness campaigns and educational initiatives (Sweden Alzheimer Europe, 2023).

These national dementia plans exemplify coordinated and systematic approaches to improve dementia diagnosis, treatment, and care in different countries. Each plan acknowledges the importance of a multidisciplinary team, public awareness, and reducing stigma to provide comprehensive and compassionate dementia care. By learning from these successful experiences, Pakistan can develop its own strategies to address the specific needs of its population while aligning with global efforts to tackle dementia on a broader scale.

Laws and regulations must be in place. To attract the attention of clinical research institutions, regulatory and legislative processes for starting clinical trials need to be streamlined and made more efficient. Pakistani practitioners should be willing to pick up novel techniques, methods, and resources in behavioral and cognitive neurology. Physicians, legislators, and nations nearby may collaborate to help Pakistan reach its goal of providing Alzheimer's patients and those caring for them with critically needed assistance (Khan, 2014). A detailed summary of the above discussion has been described in Table 1.

**Table 1:** Status of dementia care in Pakistan.

**Aspect**	**Status**
Public awareness	Limited public awareness and understanding of dementia.
Healthcare system	Dementia care is given low priority within the healthcare system.
Infrastructure	Limited specialized dementia care facilities and resources.
Caregiver support	Insufficient formal support and resources for caregivers of dementia patients.
Education and training	Lack of comprehensive dementia training programs for healthcare professionals.
Diagnosis and treatment	Limited access to early diagnosis and no specific cure for dementia.
Government policies	Absence of dedicated governmental policies for dementia care.
Research and resources	Limited research initiatives and resources dedicated to dementia in Pakistan.

## Conclusion

Dementia, a condition that includes an entire range of neurological symptoms, is increasingly becoming a burden on the healthcare system of Pakistan. As an LMIC, there is a substantial lack of resources needed to deal with this epidemic, such as daycare centers, assisted living, group therapy, etc. This is unfortunate since, with no recognized cure for the condition, patients and their families need official support facilities to enhance their living standards. Familial and informal caretakers require training and awareness sessions to better understand the needs of patients. Establishing public support services is necessary to ensure that people's financial circumstances do not affect their ability to obtain healthcare.

Clinicians and relevant healthcare professionals are unfortunately not as well-versed in the field of dementia as they ought to be, due to the inadequacy of curriculum and absence of training programs. Furthermore, the number of trained doctors is depressingly low compared to the number required to serve the entire country. The situation in the country calls for research to be conducted far more vigorously since there are a lot of gaps in knowledge specific to the nation. Research occurring in other parts of the world, most often in higher-income countries, can be modified to fit the context of Pakistan. Knowledge can be applied, provided all aspects of culture are considered with due diligence.

Primarily, time and effort need to be invested into raising awareness about the condition and removing misconceptions among the public and healthcare staff. This can take the form of social media campaigning to more structured seminars targeted at healthcare professionals. Efforts then need to be directed toward setting up nationwide policies, laws, and systems to provide affected individuals the care they deserve, and to push Pakistan toward becoming more proactive in the global research community.

## Data availability statement

The original contributions presented in the study are included in the article/supplementary material, further inquiries can be directed to the corresponding author.

## Author contributions

SA and AN were involved in the study concept, the collection of the data, drafting, literature review, data validation, supervision, and editing of the manuscript. MZ and TF were responsible for the literature review and revising of the manuscript for important intellectual content. All authors contributed to the article and approved the submitted version.
